# Experience with Pneumonia

**Published:** 1891-10

**Authors:** I. Dever

**Affiliations:** Clinton, N. Y.; Bureau of Clinical Medicine, I. H. A.


### EXPERIENCE WITH PNEUMONIA.

I. DEVER, M. D., CLINTON, N. Y.

(Bureau of Clinical Medicine, I. H. A.)

CASE I.—Wm. H., aged sixty-five, painter by trade, but drunkard by long and repeated habit. Sent for me January 12th, 1891. I found him laboring under the following abnormal conditions. Pulse one hundred and thirty per minute, stupid, restless, no inclination to talk or answer questions, his only answer to my interrogations was "I am sick, and if you can do any thing for me I want you to do it right away at that."

His friends, or those who had a right to know, informed me that he was just recovering from a grand, long, "big drunk," consequently I prescribed one dose of Nux-vom$^{2c}$ and left him to his dreams.

I called next morning, January 13th, when I learned that he had not rested during the night, but had been up frequently on account of a diarrhœa which was then dirty brown, and very offensive. He did not complain of pain in the bowels; but pressure on the abdomen, which was bloated, brought forth expressions of pain. The pulse one hundred and thirty-five; temp. one hundred and three. The cough at this time was tearing—and attended with dyspnœa likewise bloody expectoration. The tongue presented the well-known tip. The nervous system was deeply involved, as shown by the nervous restlessness, the muttering delirium and constant picking at the bed and imaginary things. Rhus$^{2m}$, one dose and Sac-lac. for a week greatly improved the case, as the fever was about all gone. The diarrhœa subsided in twenty-four hours after the first dose of medicine, but the stomach remained weak and sensitive. A small portion of brandy was offered, and no sooner swallowed than it returned. Phos.$^{1600}$, one dose, followed by Sac-lac. was all the medicine necessary to bring about his usual state of health and vigor.

CASE II.—February 7th, 1891, I was called to visit a stalwart

Hibernian. He had a dry cough with great pain in the apex of the left lung. His thirst was for cold water, everything tasting flat to him, except water. The fever had followed a severe chill—the result of a sudden change from a high degree of heat to a low temperature. Fear was pictured on every feature of his countenance, and the first words he spoke to me were, "Doctor, I shall die. I am a very sick man and I know I shall die." Acon.$^{2c}$ in water, a teaspoonful every half-hour, was given until he began to perspire freely, when Sac-lac. was substituted and continued to the end of the cure.

CASE III.—Mrs. E., aged twenty-six, the mother of two children, a slight and slender woman who presented marks of inborn scrofula, was taken sick with a slight fever and sore throat, which gradually extended to the apex of the left lung. She was subject to nightly aggravations of tickling cough attended by almost complete aphonia. Her strength, to use her own expression, had all left her, and to add to her discomfort she was unable to lie on the left side. Phos.$^{2c}$, dissolved in water, a dose every two hours, with directions if worse to stop the medicine, and if better to stop, brought about a favorable result, as it relieved the aphonia and caused the expulsion of a membrane which was the exact shape of the glottis. I gave her Sac-lac. for a week, when I discharged her as cured, or at least sufficiently cured to take charge of her household affairs.

To the older members of the International Hahnemannian Association, this paper may appear as one out of season, for have we not all prescribed in like manner for—lo! these many years? To such I would say—go higher—continue as you no doubt will in well-doing—but it is the inexperienced members of this Association that I wish to impress with the important fact that notwithstanding I have presented three clinical cases, all of which were different so far as constitution and symptoms were concerned, yet all were acute cases of sickness and all were cured by the single, similar, high remedy, prescribed with reference to a law which is universal in its action and admits of no exception in the healing art.

